# Performance of deep learning technology for evaluation of positioning quality in periapical radiography of the maxillary canine

**DOI:** 10.1007/s11282-021-00538-2

**Published:** 2021-05-26

**Authors:** Mizuho Mori, Yoshiko Ariji, Motoki Fukuda, Tomoya Kitano, Takuma Funakoshi, Wataru Nishiyama, Kiyomi Kohinata, Yukihiro Iida, Eiichiro Ariji, Akitoshi Katsumata

**Affiliations:** 1 Department of Oral Radiology, Asahi University School of Dentistry, 1851 Hozumi, Mizuho-city, Gifu 501-0296 Japan; 2 Department of Oral and Maxillofacial Radiology, Aichi-Gakuin University School of Dentistry, Nagoya, Japan

**Keywords:** Deep learning, Segmentation, Periapical radiography, Maxillary canine, Artificial intelligence

## Abstract

**Objectives:**

The aim of the present study was to create and test an automatic system for assessing the technical quality of positioning in periapical radiography of the maxillary canines using deep learning classification and segmentation techniques.

**Methods:**

We created and tested two deep learning systems using 500 periapical radiographs (250 each of good- and bad-quality images). We assigned 350, 70, and 80 images as the training, validation, and test datasets, respectively. The learning model of system 1 was created with only the classification process, whereas system 2 consisted of both the segmentation and classification models. In each model, 500 epochs of training were performed using AlexNet and U-net for classification and segmentation, respectively. The segmentation results were evaluated by the intersection over union method, with values of 0.6 or more considered as success. The classification results were compared between the two systems.

**Results:**

The segmentation performance of system 2 was recall, precision, and F measure of 0.937, 0.961, and 0.949, respectively. System 2 showed better classification performance values than those obtained by system 1. The area under the receiver operating characteristic curve values differed significantly between system 1 (0.649) and system 2 (0.927).

**Conclusions:**

The deep learning systems we created appeared to have potential benefits in evaluation of the technical positioning quality of periapical radiographs through the use of segmentation and classification functions.

## Introduction

Periapical radiography is a basic examination method for diagnosis of dental diseases including caries and periapical lesions, and the bisecting or parallel techniques with orthoradial projection are generally used in clinics. Therefore, dental students and clinical residents should initially acquire this technique and completely comprehend the causes of technical failures. Moreover, they should know that the difficulty of the periapical technique differs depending on the target sites, and the maxillary canine is one of the most frequently failed teeth [1] that is usually radiographed using the bisecting procedure in Japan, as the parallel technique cannot be applied because of the relative shallowness of the palate. To improve this technique, the ability to evaluate radiographs taken by themselves is fundamentally important. A computer-aided system to automatically evaluate the quality of radiographs could have a role in self-evaluation. Furthermore, such a system would be useful for reducing the efforts of teaching staff, who assess large numbers of radiographs taken by dental students, to achieve objective and consistent evaluations [2].

In recent years, progress in computer capacity has enabled us to apply deep learning (DL) techniques to the medical and dental fields, and it has been reported to be effective in many applications in the field of oral and maxillofacial radiology, including classification of maxillary sinusitis [3], object detection of jaw cysts/tumors [4] and maxillary sinus lesions [5], and segmentation of teeth [6, 7] and the mental foramen [8]. Regarding periapical radiographs, several applications have been reported, including automatic film mounting [9], teeth detection and numbering [10], and segmentation of teeth and lesions [11].

Many aspects of the positioning quality of periapical radiographs are frequently evaluated, such as horizontal and vertical projection angles; the position settings of receptors, such as film and imaging plates, cone-cutting, receptor distortion or bend, and mis-setting of the front and back sides of the receptor [1, 12–16]. An experienced oral and maxillofacial radiologist can take all these factors into account and instantly assess radiographs and categorize them into good or bad quality. The classification function of DL systems might provide an effective replacement for this process. In addition, a semantic segmentation technique might contribute because a tooth is generally located among three or four teeth displayed on a periapical radiograph.

Our goal is to create a fully automatic system for evaluating the positioning quality of periapical radiographs. In the present study, we created two DL systems to achieve this goal. One system was created using data without segmentation and directly classified the radiographs as good or bad quality. The other included a segmentation step before classification. The aim of the present study was to verify the created systems’ performance at evaluating the positioning quality of periapical radiographs, focusing on the maxillary canines.

## Materials and methods

This study protocol was approved by the Ethics Committee of Aichi Gakuin University (Approval No. 608), and the study was conducted in compliance with the ethical standards of the Declaration of Helsinki.

### Subjects

Periapical radiographs were taken using a GX-60 (Asahi Roentgen Industry. Co., Ltd., Kyoto, Japan) with tube voltage 60 kV, tube current 10 mA, and irradiation time 0.12–0.24 s, as recommended by the manufacturer. We used the Arcana image processing system (Cross Tech, Inc., Yokohama, Japan) with imaging plates.

Periapical radiographs containing the maxillary canines acquired from September to October 2019 were collected from the image database of Aichi Gakuin University Dental Hospital. All images of good quality were collected from the image database that stored patients’ images for clinical use. As a result, the 250 images with good quality were prepared for this study. The images of bad quality were collected by the following methods: the 150 images were collected from the image database for clinical use. The 60 images were collected from stored images taken by residents using a phantom with a dried skull embedded inside. Additional 40 images of bad quality were obtained by an author (MM) using the same phantom and image processing unit. Consequently, a total of 250 images of bad quality were prepared for the present study.

**Fig. 1:**
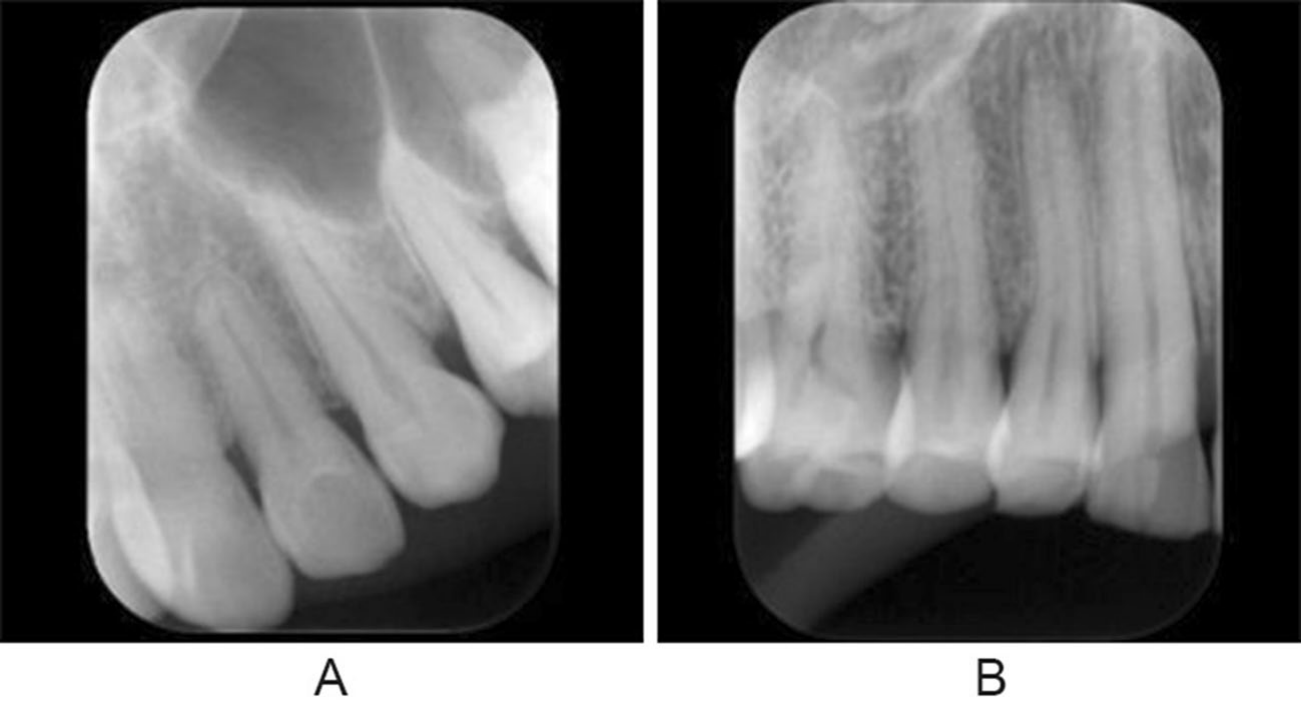
**A** Good-quality image (class 1), and **B** bad-quality image (class 0)

Two experienced radiologists (YA and AK) with more than 30 years’ experience interpreting periapical radiographs verified the image quality of the maxillary canines on the basis of the suitability of bisecting and orthoradial projection angles, the presence of cone cutting, and the appropriateness of teeth position on the images (Fig. 1). For the bisecting projection angle quality, it was assigned as good when the canine length on a radiograph was considered to be similar to actual length. It was evaluated as bad when the length was far from the actual length. For the quality of orthoradial projection angle, the quality should be ideally regarded as good when the both medial and distal proximal surfaces of the canine was not overlapped to the adjacent teeth. However, it was difficult to obtain such an image, because the canine was situated at the corner of the dental arch. Therefore, when at least one surface was not overlapped, it was assigned as good. The absence of cone cutting was good. For the teeth position, when a canine was observed approximately at the center of the image, it was evaluated as good. When all these four items were simultaneously assigned as good, the quality of radiographs were considered to have good quality. When judgments differed between the two evaluators, the final decision was made by discussion. These evaluations were used as the ground truth quality ratings.

## DL systems and their architectures

**Fig. 2:**
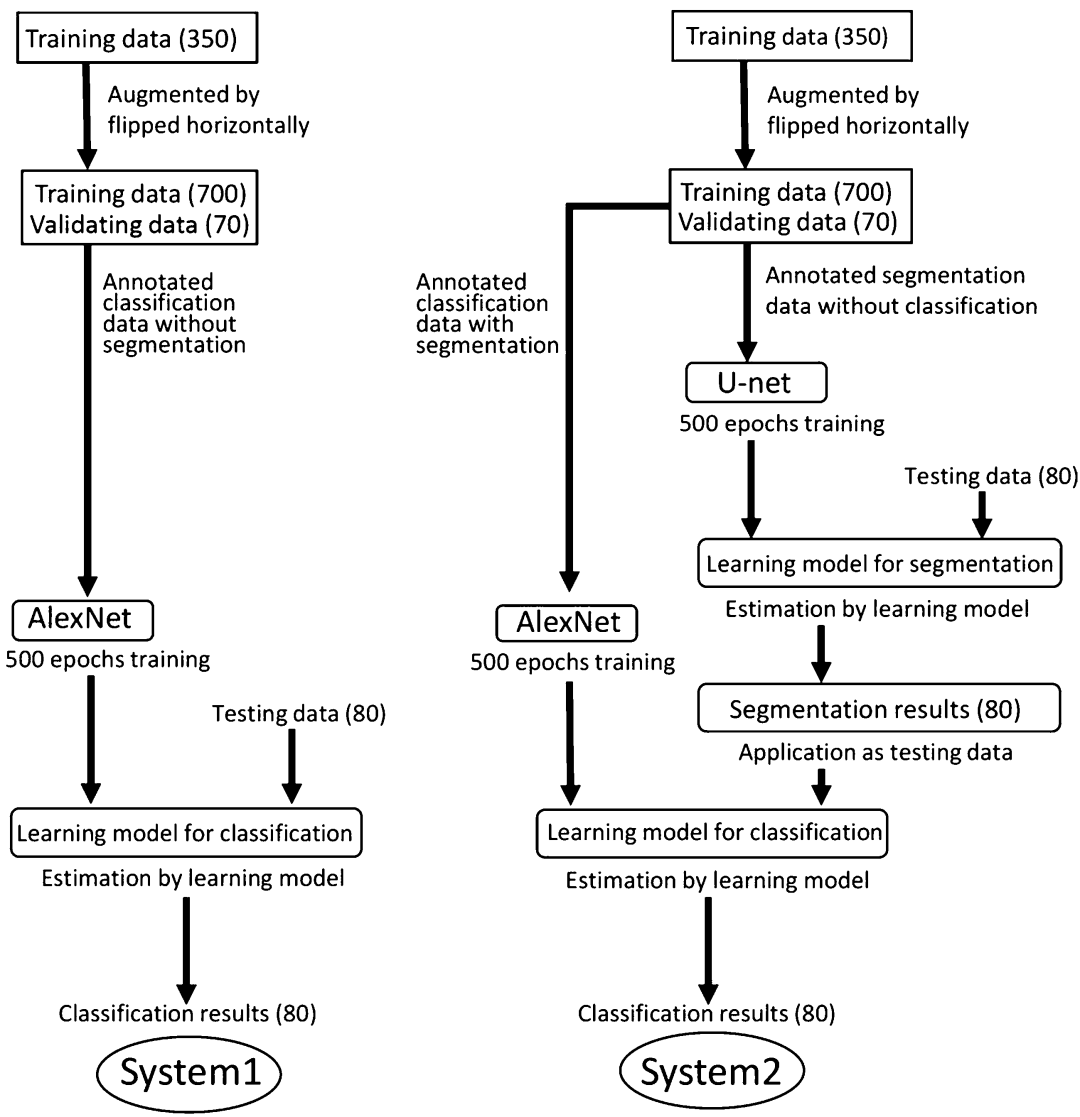
Diagram of creation of learning systems. Arabic numerals in parentheses show the number of image patches

**Fig. 3:**
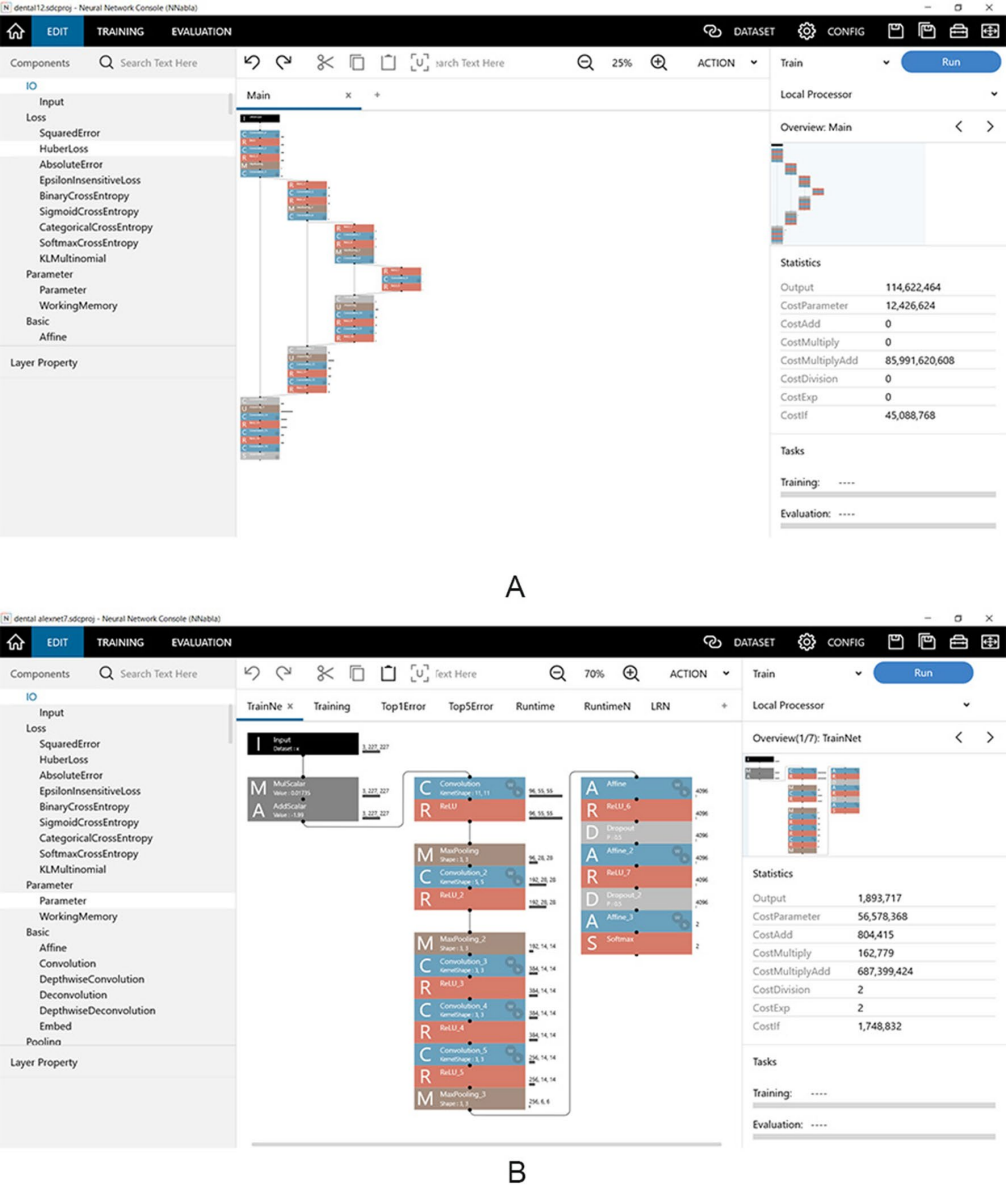
**A** and **B** are shown on a neural network console (Sony, Tokyo, Japan). **A** Uses U-net for the segmentation process, and **B** uses AlexNet for the classification process

Two DL systems were created. The first system (system 1) was developed without any segmentation process and directly classified the images as either good or bad quality (Fig. 2). The second system (system 2) initially segmented the maxillary canine on the images and thereafter classified them into the same two quality levels (Fig. 2). The DL systems were implemented on Microsoft Windows 10 with an 11-gigabyte graphics processing unit (NVIDIA GeForce GTX 1080Ti; NVIDIA, Santa Clara, CA). The neural network architectures used were U-net and AlexNet for the segmentation and classification processes, respectively, on the Neural Network Console (Sony, Tokyo, Japan) (Fig. 3).

### System 1

**Fig. 4:**
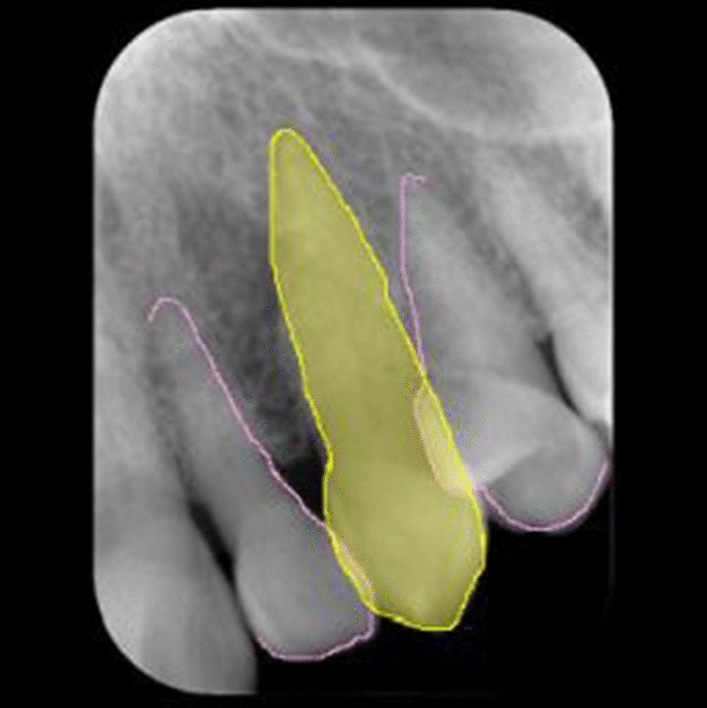
An example of an annotated image patch assigned as good quality. The left maxillary canine is painted in yellow, and the adjacent outlines of the neighboring teeth are traced in pink

The image patches were prepared in Joint Photographic Experts Group (JPEG) format with pixel resolution of 320 × 320. The long side of the intra-oral image was adjusted to 320 pixels, and the short side was positioned at the center of the 320-pixel range, with the remaining area masked as black (Fig. 4). Image patches containing 350, 70, and 80 canines were randomly assigned to the training, validation, and test datasets, respectively (Fig. 2). Each dataset contained an equal number of good- and bad-quality image patches. Annotation of the training and validation datasets was performed only for classification, with the good- and bad-quality patches assigned as class 1 and class 0, respectively. The training process was performed for 500 epochs on AlexNet.

### System 2

The images were prepared in JPEG format with a pixel resolution of 256 × 256. In the same manner as in system 1, the long side of the intra-oral image was adjusted to 256 pixels (Fig. 2). The images containing 350, 70, and 80 canines were randomly assigned to the training, validation, and test datasets, respectively (Fig. 2). Each dataset contained an equal number of good- and bad-quality images. The annotations were performed on the training and validation datasets. The maxillary canines were painted in yellow and the outlines of the adjacent teeth traced in pink using Adobe Photoshop version 21 (Adobe, Inc, San Jose, CA) (Fig. 4). The number of training images was augmented from 350 to 700 by horizontal flipping using free software (Irfan View ver.4.44; https://www.irfanview.com/). The learning process was performed for 500 epochs on U-net using the original images as the input data and the annotated images as the output data. (Fig. 2). Consequently, a learning model for segmentation was created, and thereafter, it was tested on the test data. The estimated segmentation results were outputted and used as the test data for the classification process. A learning model for classification was created based on the AlexNet using the datasets annotated in the segmentation process as good or bad quality (class 1 or 0, respectively). In this process, the images were resized to 320 × 320 pixels in JPEG format for adjustment to the network used. Similar to the segmentation process, the long side of the image was adjusted to 320 pixels. The resultant segmented images were inputted as the test dataset into the created learning model for classification.

Evaluation of model performance.

### Segmentation performance

Judgments of segmentation success were performed using the intersection over union (IoU) method. The canines on the test images were painted by an experienced radiologist (YA) as the ground truth canine areas for comparison with those predicted by the segmentation model. The IoU value was the ratio of the overlap between the predicted and ground truth areas (intersection) to the total of the two areas combined (union). These areas were determined as numbers of pixels using Adobe Photoshop version 21. When the IoU value was 0.6 or more, the segmentation was regarded to be successful, indicating a true positive.

The following indices were calculated for evaluation of segmentation performance.

- $$ {\text{Recall}} = {\text{TP}}/\left( {{\text{TP}} + {\text{FN}}} \right), $$
- $$ {\text{Precision}} = {\text{TP}}/\left( {{\text{TP}} + {\text{FP}}} \right), $$
- $$ F{\text{ measure}} = 2 \times {\text{Precision}} \times {\text{Recall}}/\left( {{\text{Precision}} + {\text{Recall}}} \right), $$

where TP is true positive, FP is false positive, and FN is false negative.

### Classification performance

The classification results estimated by the models were represented as the prediction values (probability) of the ground truth. When the value was 50% or more for good quality (class 1), the evaluation was regarded as positive. For the evaluation of classification performance, the following indicators were calculated:

- $$ {\text{Accuracy}} = \left( {{\text{TP}} + {\text{TN}}} \right)/\left( {{\text{TP}} + {\text{FP}} + {\text{TN}} + {\text{FN}}} \right), $$
- $$ {\text{Sensitivity}} = {\text{TP}}/\left( {{\text{TP}} + {\text{FN}}} \right), $$
- $$ {\text{Specificity}} = {\text{TN}}/\left( {{\text{FP}} + {\text{TN}}} \right), $$

where TP is true positive, FP is false positive, and FN is false negative.

In addition, the predictive values for positive evaluation (good quality) corresponded to the true positive fraction (sensitivity), and those for negative evaluation were the false positive fraction (1 − specificity). The receiver operating characteristic curve and area under the curve (AUC) were calculated.

The AUC values were compared between systems 1 and 2 by Chi-square test, with *p* < 0.05 being significant.

## Results

### System 1

It took 19 min 29 s to complete the 500 epochs to train the learning model, and it took 14 s to evaluate the model’s performance in the testing process.

The sensitivity, specificity, accuracy, and AUC were 0.625, 0550, 0.588, and 0.649, respectively (Table 1).

**Table 1:** Classification performances of two systems

	System 1	System 2
True positive (No. of patches)	25	37
True negative (No. of patches)	22	33
False positive (No. of patches)	15	3
False negative (No. of patches)	18	7
Sensitivity	0.625	0.925
Specificity	0.550	0.825
Accuracy	0.588	0.875
AUC	0.649*	0.927*

*Statistically significant difference with *p* ≦ 0.001 by Chi-square test

### System 2

The segmentation model took 6 h 47 min to create and 20 s to test. The classification model took 14 min 32 s to create and 17 s to test.

**Fig. 5:**
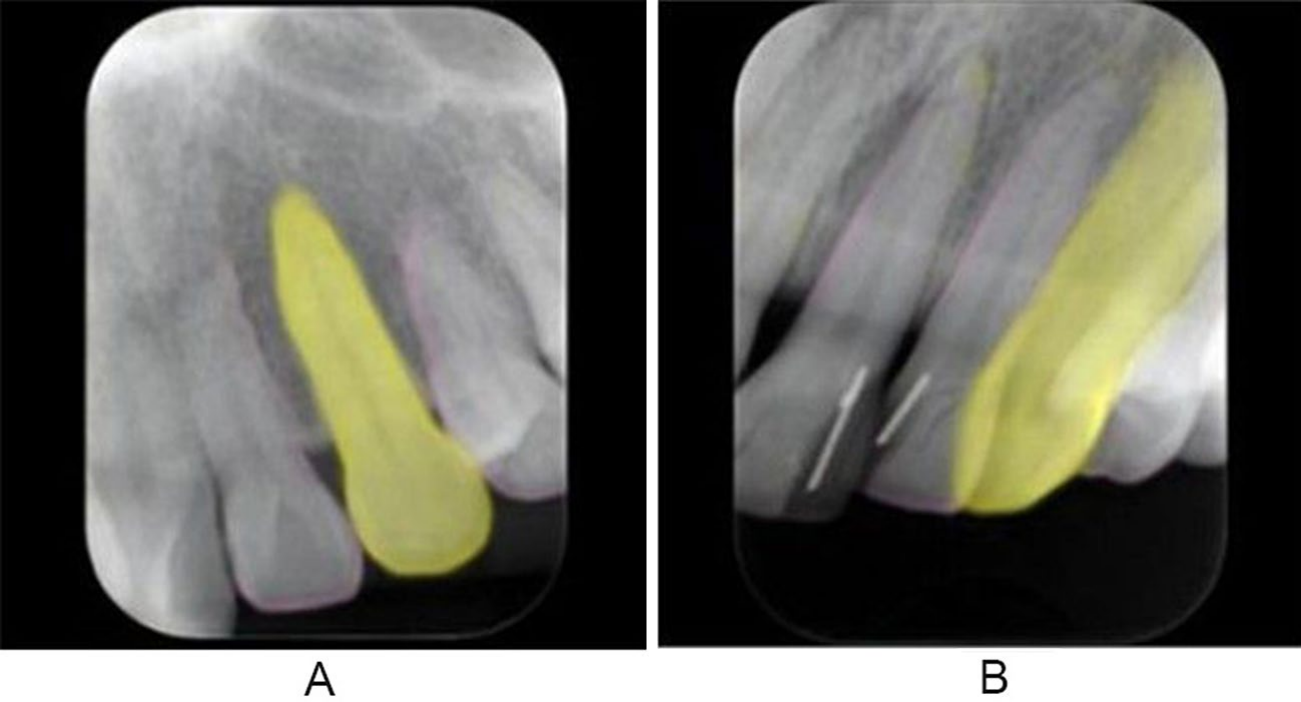
**A** and **B** show that the left maxillary canine is successfully segmented. **A** is correctly classified as a good-quality image (class 1) and **B** as a bad-quality image (class 0)

**Fig. 6:**
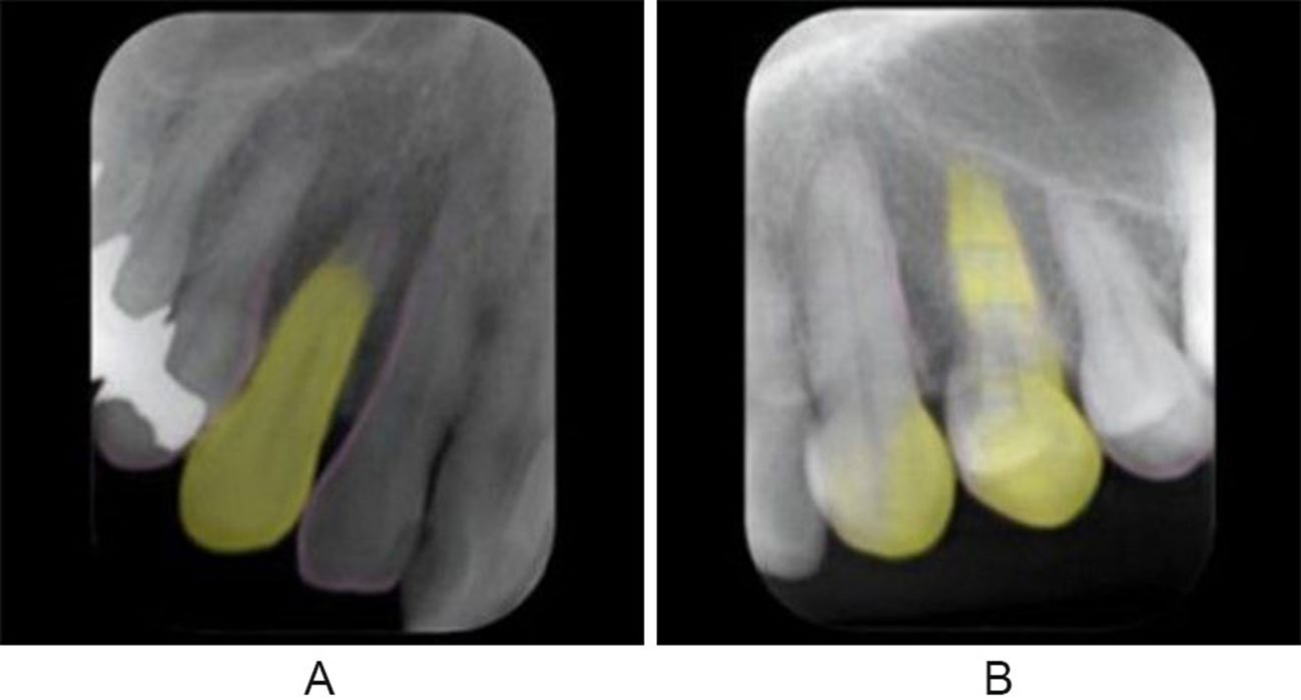
In **A**, the right maxillary canine is successfully segmented (intersection over union: 0.81) but erroneously classified; the bad quality is probably caused by shortening of the painted root area. In **B**, the left maxillary canine, for which the ground truth is bad quality, because the canine is not positioned at the center of the image, is not successfully segmented (intersection over union: 0.16). The first premolar painted in a relatively wide area is probably regarded as the canine and classified as good quality

The segmentation performance is summarized in Table 2. The canines were successfully segmented in 74 of 80 test patches (IoU ≥ 0.6) (Fig. 5). Only three areas of other teeth were falsely segmented as canines (Fig. 6). The model’s recall, precision, and F measure showed high values.

**Table 2:** Segmentation performance of system 2

True positive (No. of patches)	74
False positive (No. of patches)	3
Recall	0.925
Precision	0.961
*F* measure	0.943

**Fig. 7:**
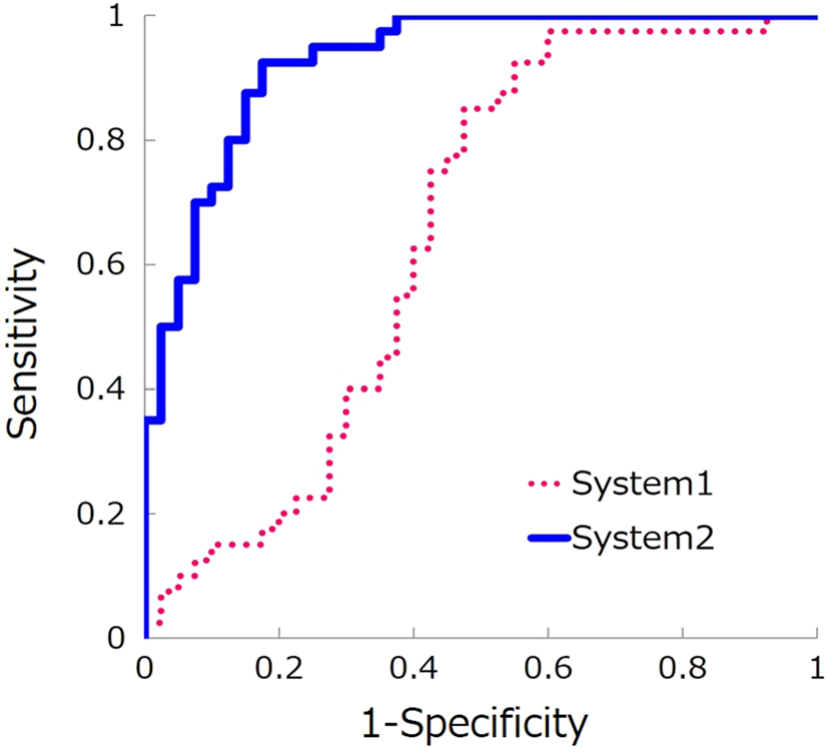
Receiver operating characteristic (ROC) curve. The continuous line denotes system 2, which was created with the segmentation process. The dotted line shows the results of system 1, which was created without the segmentation process. The AUCs were significantly different (*p* < 0.001)

For system 2’s classification performance, the sensitivity, specificity, accuracy, and AUC were 0.925, 0.825, 0.875, and 0.927, respectively (Table 1). The AUC of system 2 was significantly higher than that of system 1 (*p* ≤ 0.001) (Table 1, Fig. 7).

## Discussion

Various causes of failure have been reported in various aspects of periapical radiography with the bisecting and parallel techniques, including the suitability of horizontal and vertical projection angles, the appropriateness of receptor positioning, and the presence or absence of cone cutting [1, 12–16]. Accordingly, we comprehensively evaluated these aspects and classified images of canines into two categories to determine their ground truth quality. The exposure conditions were not taken into account, because small inadequacies could be remediated using image processing in digital systems.

As for tooth segmentation on periapical radiography, Ronneberger et al. [17] reported relatively low recall, precision, and F measure values for upper and lower molar segmentations using a U-net architecture (0.747, 0.453, and 0.564, respectively). Contrarily, the present results showed good segmentation performance (recall, precision, and F measure values of 0.937, 0.961, and 0.949, respectively). This discrepancy can be partially attributed to the differences in root configuration between the teeth, indicating the difference in the number of roots per tooth. However, there have been some reports in which all types of teeth, including the maxillary canines, were segmented on panoramic radiographs [6, 7, 18]. Leite et al. reported good performance at segmenting the maxillary canines (recall, precision, and *F* measure of 0.969, 0.964, and 0.973, respectively) [7]. Lee et al. also reported high segmentation accuracy of 0.889 for the maxillary canines [6]. In spite of the difference in the modalities used, the present results support those of the other reports about maxillary canine segmentation on radiographs.

In our previous studies evaluating the classification performance on panoramic radiographs, relatively small areas, such as those of the maxillary incisor and maxillary sinus [3, 19], were cropped from areas of entire panoramic radiographs, and good performance was verified. The learning models in those studies were created without segmentation. Therefore, model 1 was created without the segmentation process to compare its performance to that of model 2, which was created with the segmentation process. As a result, the model’s classification performance (measured as AUC) was significantly improved by including the segmentation step before classification. This means that we should try to perform segmentation before classification when classification performance would otherwise be insufficient.

Some of the classification failures observed in our dataset might have been caused by segmentation failures, as the technical quality was generally assigned as bad when a tooth other than the target canine was painted as a canine. When the root apex of the maxillary canine could not be sufficiently segmented, the DL model might have classified the image as bad quality owing to recognizing the result as shortening of the root. Therefore, the model’s classification performance could be improved if the segmentation performance could be improved.

The present study has several limitations. First, the causes of failure could not be definitely identified, because the radiographs were classified on the basis of overall suitability. For self-assessment purposes for students and residents, it is desirable to build a system that can separately clarify the causes of failure. Second, for the evaluation of large numbers of images in the field of education, false classification of truly good-quality images into the bad category should be avoided. Although the classification was performed with only two categories in the present study, three categories (i.e., good, undecided, and bad quality) might be better if the undecided images can be reevaluated by the instructors. Third, phantom images were used in addition to patient images, because there were not enough images of poor quality in the database. Although actual cause was unclear, mixing patient and phantom images might affect quality evaluation. Four, the number of datasets was too small to generalize the results, and only the canines were evaluated. In future investigations, a system that can evaluate the quality of all teeth should be developed with larger datasets including various pathologies, such as deep cares, periapical lesion and root fracture.

In conclusion, we confirmed a potential application of DL systems in the evaluation of the technical positioning quality of intra-oral radiographs using segmentation and classification techniques.

## References

[1] Peker I, Alkurt MT (2009). Evaluation of radiographic errors made by undergraduate dental students in periapical radiography. N Y State Dent J.

[2] Fujita H (2003). Current status and future on developments of computer-aided diagnosis systems for medical images. J Soc Photogr Sci Technol Jpn.

[3] Murata M, Ariji Y, Ohashi Y, Kawai T, Fukuda M, Funakoshi T (2019). Deep-learning classification using convolutional neural network for evaluation of maxillary sinusitis on panoramic radiography. Oral Radiol.

[4] Ariji Y, Yanashita Y, Kutsuna S, Muramatsu C, Fukuda M, Kise Y (2019). Automatic detection and classification of radiolucent lesions in the mandible on panoramic radiographs using a deep learning object detection technique. Oral Surg Oral Med Oral Pathol Oral Radiol.

[5] Kuwana R, Ariji Y, Fukuda M, Kise Y, Nozawa M, Kuwada C (2020). Performance of deep learning object detection technology in the detection and diagnosis of maxillary sinus lesions on panoramic radiographs. Dentomaxillofac Radiol.

[6] Lee JH, Han SS, Kim YH, Lee C, Kim I (2020). Application of a fully deep convolutional neural network to the automation of tooth segmentation on panoramic radiographs. Oral Surg Oral Med Oral Pathol Oral Radiol.

[7] Leite AF, Gerven AV, Willems H, Beznik T, Lahoud P, Gaêta-Araujo H (2021). Artificial intelligence-driven novel tool for tooth detection and segmentation on panoramic radiographs. Clin Oral Investig.

[8] Kats L, Vered M, Blumer S, Kats E (2020). Neural network detection and segmentation of mental foramen in panoramic imaging. J Clin Pediatr Dent.

[9] Kitano T, Mori M, Nishiyama W, Kohinata K, Iida Y, Fujita H (2021). Classification of intraoral X-ray images using artificial intelligence. Dental Radiol.

[10] Chen H, Zhang K, Lyu P, Li H, Zhang L, Wu J (2019). A deep learning approach to automatic teeth detection and numbering based on object detection in dental periapical films. Sci Rep.

[11] Khan HA, Haider MA, Ansari HA, Ishaq H, Kiyani A, Sohali K (2020). Automated feature detection in dental periapical radiographs by using deep learning. Oral Med Oral Pathol Oral Radiol.

[12] Nakayama M, Takada S, Kise Y, Nishiyama W, Izumi M, Ariji Y (2016). Post-graduate clinical training at the Department of Radiology and Diagnostic Imaging, Aichi-Gakuin University Dental Hospital—evaluation of technique for intraoral radiography and questionnaire survey of the training. JJDEA.

[13] Wuehrmann AH (1972). Evaluation criteria for intraoral radiographic film quality. J Am Dent Assoc.

[14] Patel JR (1979). Intraoral radiographic errors. Oral Surg Oral Med Oral Pathol.

[15] Patel JR, Greer DF (1986). Evaluating student progress through error reduction in intraoral radiographic technique. Oral Surg Oral Med Oral Pathol.

[16] Zhang W, Huynh CP, Abramovitch K, Leon IL, Arvizu L (2012). Comparison of technique errors of intraoral radiographs taken on film v photostimulable phosphor (PSP) plates. Tex Dent J.

[17] Ronneberger O, Fischer P, Brox T. Dental X-ray image segmenation using a U-shaped deep convolutional network. ISBI 2015, April 16–19.

[18] Wirtz A, Mirashi SG, Wesarg S. Automatic teeth segmentation in panoramic x-ray images using a coupled shape model in combination with a neural network. In: MICCAI 2018 Proceedings, pp 712–19; 10.1007/978-3-030-00937-3_81.

[19] Kuwada C, Ariji Y, Fukuda M, Kise Y, Fujita H, Katsumata A, Ariji E (2020). Deep learning systems for detecting and classifying the presence of impacted supernumerary teeth in the maxillary incisor region on panoramic radiographs. Oral Med Oral Pathol Oral Radiol.

